# Decoding Amyotrophic Lateral Sclerosis: Discovery of Novel Disease-Related Biomarkers and Future Perspectives in Neurodegeneration

**DOI:** 10.1155/2014/629630

**Published:** 2014-08-04

**Authors:** Ana Cristina Calvo, Pierre-François Pradat, Deise M. F. Mendonça, Raquel Manzano

**Affiliations:** ^1^LAGENBIO-I3A, Veterinary Faculty of Zaragoza, Aragonese Institute of Health Sciences (IACS), University of Zaragoza, Miguel Servet 177, 50013 Zaragoza, Spain; ^2^AP-HP, Hôpital Pitié-Salpêtrière, Département des Maladies du Système Nerveux, 75005 Paris, France; ^3^Sorbonne Universités, UPMC Univ Paris 06, INSERM, CNRS, Laboratoire d'Imagerie Biomédicale, 75005 Paris, France; ^4^Biosciences Department, Laboratory of Neurobiology of Degenerative Diseases of the Nervous System, Federal University of Sergipe, Avenue Vereador Olimpio Grande s/n, Centro, 49500-000 Itabaiana, SE, Brazil; ^5^University of Oxford, Wellington Square, Oxford OX1 2JD, UK

Amyotrophic lateral sclerosis (ALS) belongs to the group of motor neuron diseases, in which the degeneration, the weakness of voluntary muscles, and death of motor neurons gradually spread along disease progression. ALS comes from Greek language and means “no muscle nourishment” (“*amyotrophic*”), “area of the spinal cord where affected nerve cells are localized” (“*lateral*”), and “the degeneration and hardening of the spinal cord” (“*sclerosis*”). The fundamental contributions of the celebrated neurologist Jean-Martin Charcot at the end of the nineteenth century provided the first description of ALS. His neurological work was based on the “anatomo-clinical method,” in which he determined the correlation between clinical signs detected during life and anatomical lesions seen at death. Nowadays, the pathophysiological mechanisms that prompt the neurodegenerative process in both forms of the disease, familial and sporadic ALS, still remain to be elucidated. However, there is growing evidence that the pathogenic process involved in ALS is multifactorial and includes oxidative stress, glutamate excitotoxicity, mitochondrial dysfunction, axonal transport systems, and dysfunction of glial cells, yielding the damage of critical proteins and organelles in the motor neurons and muscles and triggering the neurodegeneration. In this complex scenario, the need for identifying potential biomarkers that could make an earlier prognosis and diagnosis of the disease possible becomes an essential step to finally decode the molecular basis of ALS and to provide promising therapeutic strategies.

This special issue provides more in-depth information, not only considering relevant molecular and plastic features in ALS but also including accurate reviews focused on potential biomarkers of the disease in different tissues. More specifically, the role of two molecular factors, the vascular endothelial growth factor (VEGF) and the glial cell-derived neurotrophic factor (GDNF), is described. In particular, detailed characterization of VEGF in different human tissues and its connection to a potential therapeutic approach for ALS is addressed. Regarding GDNF, recent findings could pave the way for further studies dealing with its receptor, c-Ret, and its possible connection to the enteric nervous system in ALS human samples. Following with the molecular characterization of the disease, the involvement of dorsal root ganglion sensory neurons under misfolded SOD-1 mediated neurotoxicity is described in a murine model of ALS. In line with the neuronal plasticity in the spinal cord from G93A mice, neuronal progenitor cells differentiation towards a neuron-like phenotype under lithium treatment is reported.

A novel 3D mechanoelectrochemical model that can predict and quantify the impact of immobilized cartilage from ALS patients suffering a progressive loss of motion is documented in this issue. Particularly interesting is the use of neuroimaging techniques to provide an accurate identification of potential prognostic and diagnostic biomarkers of the disease at any stage. In this regard, a detailed review about neuroimaging studies in ALS, especially magnetic resonance imaging studies and spinal cord neuroimaging, is presented.

Last but not least, a careful revision of novel biomarkers, such as aquaporin-4, AQP4, and the inwardly rectifying potassium channel Kir4.1, and their role in the maintenance of ion homeostasis at the blood-brain barrier in ALS animal models and patients is addressed. A more extended revision focused on a wide-ranging panel of biomarkers in blood is described based on the different pathological mechanisms that can contribute to the progression of the disease. Finally, a general update in ALS focusing on the potential ALS biomarkers identified along disease progression in animal models and ALS patients is also included.

We hope that the accurate and updated manuscripts in this issue will provide not only new insights into the search of reliable biomarkers but also a starting point for innovative perspectives in the disease.

*Ana Cristina Calvo* *Pierre-François Pradat* *Deise M. F. Mendonça* *Raquel Manzano*

